# Mechanical Properties of Glioblastoma: Perspectives for YAP/TAZ Signaling Pathway and Beyond

**DOI:** 10.3390/diseases11020086

**Published:** 2023-06-14

**Authors:** Bruno Pontes, Fabio A. Mendes

**Affiliations:** 1 Instituto de Ciências Biomédicas, Universidade Federal do Rio de Janeiro, Rio de Janeiro 21941-902, RJ, Brazil; 2 Centro Nacional de Biologia Estrutural e Bioimagem (CENABIO), Universidade Federal do Rio de Janeiro, Rio de Janeiro 21941-902, RJ, Brazil

**Keywords:** glioblastoma, mechanobiology, mechanotransduction, hippo signaling pathway, YAP, TAZ, tumor progression

## Abstract

Glioblastoma is a highly aggressive brain tumor with a poor prognosis. Recent studies have suggested that mechanobiology, the study of how physical forces influence cellular behavior, plays an important role in glioblastoma progression. Several signaling pathways, molecules, and effectors, such as focal adhesions, stretch-activated ion channels, or membrane tension variations, have been studied in this regard. Also investigated are YAP/TAZ, downstream effectors of the Hippo pathway, which is a key regulator of cell proliferation and differentiation. In glioblastoma, YAP/TAZ have been shown to promote tumor growth and invasion by regulating genes involved in cell adhesion, migration, and extracellular matrix remodeling. YAP/TAZ can be activated by mechanical cues such as cell stiffness, matrix rigidity, and cell shape changes, which are all altered in the tumor microenvironment. Furthermore, YAP/TAZ have been shown to crosstalk with other signaling pathways, such as AKT, mTOR, and WNT, which are dysregulated in glioblastoma. Thus, understanding the role of mechanobiology and YAP/TAZ in glioblastoma progression could provide new insights into the development of novel therapeutic strategies. Targeting YAP/TAZ and mechanotransduction pathways in glioblastoma may offer a promising approach to treating this deadly disease.

## 1. Introduction

Cancer is a disease initiated by genetic modifications that cause uncontrolled division of abnormal cells, creating a tumor cell mass with the ability to spread to other organs. Such tumor masses develop by altering their biochemical and physical contexts [1]. Much has been described about the biochemical changes [2], but less is known about the mechanical modifications in cancer cells and tissues, as well as their consequences for cancer progression [3].

Looking at the macroscopic level, cancer tissue, in general, not only senses but also induces numerous physical changes in its surrounding environment. Such alterations include aberrant tissue architecture, altered material properties, increased solid stress, and elevated interstitial fluid pressure (IFP) [3]. The most easily detectable change in cancerous tissue is its increase in stiffness, which often occurs due to an increase in both the number of cells and the deposition of extracellular matrix (ECM) proteins by the tumor and its surrounding tissues. As the tumor grows, it also causes deformation and displacement of neighboring structures, leading to tensile and compressive forces that generate solid stress nearby [4]. As a consequence, it disrupts normal tissue structure and collapses blood and lymphatic vessels inside the tumor and adjacent stroma, compromising blood flow and lymphatic drainage, which leads to fluid accumulation and elevated IFP [5]. The increase in IFP, in turn, facilitates the flow of interstitial fluid from the tumor to the surrounding non-cancerous tissues, transporting tumor-secreted factors to other regions [5].

At the microscopic level, tumor and stroma cells respond to physical cues through a process known as mechanotransduction, by which mechanical inputs are converted into biochemical signals [6]. Several mechanisms by which tumor and stroma cells sense physical cues have been described [7]. One of the most studied is through focal adhesions [8]. Focal adhesions are complexes of multiple proteins formed by clusters of transmembrane integrin heterodimers [9] in the plasma membrane that bind to ECM proteins, such as collagen, fibronectin, and laminin, and also connect to the actin cytoskeleton, using scaffolding proteins, such as talin and vinculin [10]. Thus, changes in external mechanical forces, usually caused by modifications in protein expression and in the structural organization of cancer cell ECM, are immediately transmitted to focal adhesions and cause alterations in their conformational proteins, e.g., talin, thereby recruiting downstream signaling molecules that elicit biochemical responses [10]. Similarly, cell-to-cell adhesions formed between transmembrane cadherins that bind to the actin cytoskeleton through catenin proteins [11] or intermediate filament-based complexes, such as desmosomes [12], become modified due to increase in number of cells and also induces changes in the propagation of applied forces [12]. Altogether, the modifications described can act on tumor and stroma cells, culminating with changes in the cytoskeleton that can impact the nucleus architecture via the LINC complex proteins [13]. The resulting deformation of the nucleus can alter chromatin packing and transport of biomolecules through nuclear pores, promoting or inhibiting the transcription of mechanoresponsive genes [13]. In addition to cytoskeleton-dependent mechanisms, stretch-activated ion channels, such as Piezo1, transport ions from the extracellular space into the cytosol upon changes in membrane tension [14,15,16], widely described as being modified in tumor/migrating cells [17], and eliciting biochemical responses through altered intracellular ion concentrations and membrane potentials. Interestingly, changes in membrane tension can also modulate cell signaling by unfolding or refolding membrane invaginations [17,18] and controlling integrin-based focal adhesions at the leading edge of cells [19]. Finally, also investigated are YAP and TAZ, downstream effectors of the Hippo pathway, and key regulators of cell proliferation and differentiation [20]. It is already known that YAP and TAZ can be activated by mechanical cues such as cell stiffness, matrix rigidity, and cell shape, all altered in the tumor microenvironment [21].

Among the most studied types of cancer is glioblastoma (GBM), a highly malignant primary brain tumor with presumed origin from astrocytic cells. It is the most common and aggressive form of primary brain tumor in adults, accounting for approximately 50% of all gliomas and 15% of all primary brain tumors [22]. Despite intensive therapeutic interventions, including surgery, radiotherapy, and chemotherapy, the prognosis remains poor, with a median survival of 15 months and a five-year survival rate of less than 10% [23]. Studies have shown that GBM is an immensely heterogeneous tumor. The tumor mass can be divided into two large regions: a necrotic center and a border containing cells with a high migratory capacity [24]. As GBM develops, the size of the brain gradually expands, leading to an increase in intracranial pressure (ICP), which is typically around 17–19 mmHg but can rise to 25 mmHg in later stages of GBM progression, resulting in direct mechanical stresses and solid forces between the brain tissue and skull [3,25]. Additionally, the water content inside the brain slightly increases during GBM progression [26,27]. Such a phenomenon is associated with ECM alterations and leads to cerebral edema or brain swelling [26,27].

GBM is not just composed of highly proliferative malignant cells, but also immune cells and infiltrating stromal cells, vascular endothelial cells, and pericytes, which all form distinct niches in the tumor. Within these niches, different tumor cell types (proliferating, infiltrating, cancer stem cell-like) and various non-cancerous cells (microglia, macrophages, dendritic cells, lymphocytes) dynamically shape different parts of the tumor. Such diversity results in different microenvironments within the tumor, ranging from solid tumor cores with densely packed proliferating cells to necrotic areas, perivascular areas around vessels with endothelial proliferation, and hypoxic regions [22,24], as well as cells expressing different stem markers, the so-called tumor-initiating cells or tumor stem cells [24]. Therefore, a better understanding of the underlying genetic and molecular mechanisms of GBM progression must also take into account different mechanobiological integrated experimental approaches across biological and physical sciences. Developing a better understanding of such mechanisms may pave the way for the discovery of novel therapeutics for this very aggressive type of central nervous system tumor.

The present review examines the mechanobiological changes occurring in the tissue microenvironment that drive GBM progression, although the concepts discussed are relevant to most other solid tumors. We revise several signaling pathways, molecules, and effectors involved, such as focal adhesions, stretch-activated ion channels, and membrane tension variations. We also focus on YAP/TAZ mechanosignaling and their consequences for GBM progression.

## 2. Mechanics of GBM

While the majority of cancer cell invasion is primarily guided by structural cues from the ECM, GBM invasion is guided by the unique architecture and structural characteristics of the brain’s parenchyma together with its vasculature. In vivo studies using GBM mouse models have demonstrated that tumor cells associated with blood vessels and white matter tracts have higher net displacements than cells not associated with these structures, suggesting that they promote infiltration [28,29,30]. Vascular structures and white matter tracts facilitate invasion due to their divergence with the surrounding parenchyma, comprised of an isotropic ECM consisting mainly of four components: (i) glycosaminoglycans such as keratin sulfate, heparin sulfate, dermatan sulfate; (ii) proteoglycans such as aggrecan, brevican, glypican-1, versican, and tenascin-C [31]; (iii) fibrillary glycoproteins such as collagen, fibronectin, and laminin; and (iv) several growth factors [31]. In contrast, white matter tracts are surrounded by a linearized ECM and, together with blood vessels, which similarly provide a linear geometry, preferentially allow GBM cell migration [31].

As GBM progression proceeds, there is an overexpression of certain brain ECM components that cause ECM stiffening [1,2,31]. In particular, the overexpression of Hyaluronic acid (HA), collagen, tenascin-C, fibronectin, and brevican within the GBM ECM all contribute to the stiffening process [32]. During GBM development, the stiffness of normal brain ECM (0.2 to 1.2 kPa) can increase up to 45 kPa, which activates the mechanotransduction process in GBM cells [33,34,35]. Such ECM-stiffening capacity to activate mechanosignaling processes can independently stimulate GBM aggression, contributing to GBM recurrence even in IDH1-mutant lower-grade glioma cells via a tension-dependent positive feedback loop mechanism that reduces miR-203 suppression of HIF1α, stimulating tenascin-C expression [36].

Overexpression of HA also significantly alters the mechanics of the brain tissue [37], while increased collagen expression promotes aligned microarchitecture in the ECM structure [38]. Fibronectin expression also increases and promotes cell adhesion properties [39]. Furthermore, an altered ECM in GBM facilitates matrix metalloproteinase (MMP) activity, which degrades ECM proteins and weakens the ECM’s mechanical properties, opposing the stiffening phenomena [40]. Consequently, the structure of the ECM changes, forming a confined space with an aligned microarchitecture [41,42]. Such modifications resemble the linear structure of white matter tracts and blood vessels and thus promote a later increase in the migratory and invasive states of GBM cells [41,42].

As the brain ECM stiffness increases, it activates mechanosensors in GBM cells, which transmit forces via chemical signals [9,43]. However, it is crucial to note that GBM cells often develop abnormalities in their mechanosensory machinery, including abnormal expression of important molecular components. Focal adhesion kinase (FAK), a critical mechanosensory protein, is often overexpressed in many GBM tumors [44]. Moreover, GBM frequently exhibits a modified expression of integrins, which are crucial for the physical transmission of force from the actin cytoskeleton to the extracellular matrix and for mediating attachment to the ECM [45,46,47]. Increased ECM stiffness is known to stimulate the formation of focal adhesion complexes in GBM cells, which are rich in integrin adhesion receptors and play a crucial role in bidirectional transmembrane communication. The dynamic assembly and disassembly of focal adhesions are central to cell migration, with both the composition and morphology of the focal adhesion continuously reorganizing [48].

F-actin is another key component in GBM mechanotransduction [34,49] and is greatly influenced by mechanical stress [3]. Integrins at focal adhesion sites enable F-actin to detect the stiffness of the extracellular matrix, which can then modulate its polymerization. Upon activation of several mechanosensors, cell cytoskeleton remodeling rose as the hallmark of the adaptive response of GBM cells to ECM alterations and applied mechanical stresses [3,50,51]. The remodeling of the cytoskeleton favors GBM invasiveness and facilitates its migratory state [50].

As GBM cells infiltrate the brain tissue, they employ various modes of motility that depend on the local microenvironment. These modes differ in speed and molecular mechanisms, but all involve adhesion and force transmission between the cytoskeleton and ECM. CD44, an adhesion receptor for HA, is crucial for GBM cells to navigate through the brain parenchyma [52,53]. CD44 allows GBM cells to engage and transduce mechanical signals from HA [54]. Studies have shown that the interaction between CD44 and HA contributes to GBM pathogenesis by promoting tumor cell survival, proliferation, and invasion. For instance, CD44-HA interaction activates Rho GTPases, leading to the remodeling of the actin cytoskeleton and the formation of cell protrusions, such as invadopodia, that facilitate tumor cell invasion [55]. Moreover, CD44-HA interaction activates downstream signaling pathways, such as the PI3K/Akt and MAPK/ERK, which promote tumor cell proliferation and survival [55].

Apart from cytoskeletal changes involved in GBM proliferation, survival, and invasion, Chen and collaborators [56] have identified a crucial mechanosignaling pathway that involves the Piezo1 ion channel, overexpressed in various types and grades of cancer, including GBM. The authors have shown that the Piezo1 channel is necessary for tumor growth in vitro and in vivo. In mouse models, inhibiting the ion channel has resulted in significantly longer survival and reduced tumor growth [56]. Further investigation revealed that Piezo1 acts as a central hub in a mechanotransduction cascade, with the channel located at sites of focal adhesion. Moreover, the same authors have also demonstrated a feed-forward mechanism, whereby Piezo1 regulates other ECM remodeling genes, including TAZ and FHL3. In experiments with Piezo1 knockdown, stiffness-dependent GBM cell growth was not observed. Additionally, in experiments using varying stiffness hydrogels to assess the stiffness-dependent growth of GBM cells, increased expression of Piezo1 was observed [56].

Overall, our understanding of how mechanical stimuli are transduced in GBM is still limited, but recent progress revealed some insights into the interactions between the microenvironment and cellular networks that underlie the effects of mechanical properties on disease progression. It is clear now that mechanical stimuli transduction in GBM can be broadly classified into those involving mechanosensitive ion channels and those involving non-ion channel-based mechanotransduction; the latter includes a complex array of poorly understood signaling pathways such as integrin signaling, ligand-mediated signaling through interaction with ECM components, receptor-mediated signaling through interaction with ECM components [57], the Hippo signaling pathway (better detailed in the following sections) and, much less understood, the effects of membrane tension changes [17,19] in inhibiting cancer cell migration and invasion [18].

## 3. The Hippo Signaling Pathway

The Hippo signaling pathway controls the balance between cell proliferation and apoptosis, and this balance is critical for tissue homeostasis at different stages of embryonic development, in aging processes, and in several diseases. In embryonic tissues, during development, it acts by controlling the correct size of organs and, when uncontrolled, can lead to tumorigenic processes. The first components of the Hippo signaling pathway were cloned and characterized in the 1990s [58]; however, it was only in the early 2000s that its signaling cascade began to be unraveled [20,59,60] and its functions more clarified.

**Figure 1:**
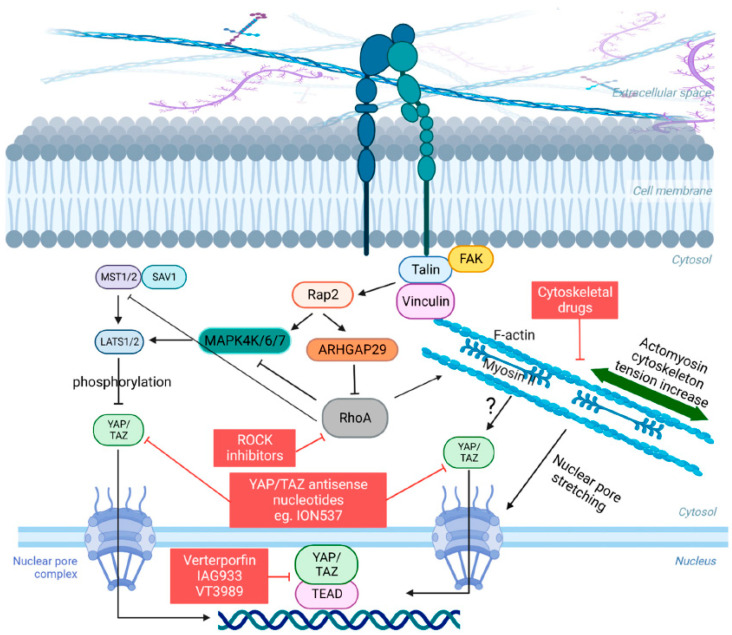
Schematic of the Hippo pathway and YAP/TAZ mechanosignaling. When cells encounter a rigid ECM, they respond by adjusting their cytoskeletal tension through integrin-mediated focal adhesions, which link the ECM to the intracellular F-actin cytoskeleton, involving important kinases such as FAK and other adhesion proteins such as Vinculin and Talin. This process leads to an increase in actomyosin tension and a reorganization of the cytoskeleton. Key components such as RhoA and myosin II motors are also required for this tension-mediated restructuring. The modulation of YAP/TAZ nuclear-cytoplasmic shuttling occurs in response to actomyosin tension, which transduces physical cues into cellular signals. The F-actin cytoskeleton also influences the mechanical properties and shape of the nucleus, promoting the nuclear entry of YAP/TAZ by inducing nuclear deformation, facilitated by the stretching of nuclear pore complexes. The Hippo pathway acts as a negative regulator of YAP/TAZ by directly phosphorylating these proteins through LATS1/LATS2 or indirectly by influencing the actin cytoskeleton. Conversely, actomyosin contractility can impact the activity of LATS1/LATS2 through the GTPase RAP2. Finally, compounds that are capable of influencing some stage of this intricate signaling pathway are highlighted in red squares.

The Hippo pathway gets its name from the effect of mutations in this gene in *Drosophila melanogaster*. Knockout flies for Hippo showed tissue overgrowth. Deletion of the Hippo gene caused an increase in proliferation and a decrease in apoptosis by increasing the expression of the target genes cyclin E and the inhibitor of cell death, diap1 [20]. The Hippo protein in humans is MST 1/2, a serine-threonine kinase that complexes with the Salvador homolog 1 (SAV1) protein and phosphorylates LATS 1/2 protein [59]. When phosphorylated, LATS 1/2 kinases phosphorylate the Hippo pathway effectors, YAP (Yes-associated protein), and TAZ (Transcriptional activator with PDZ-binding motif). Phosphorylated YAP and TAZ remain retained and inactive in the cytoplasm. However, when dephosphorylated, YAP and TAZ go to the nucleus, where they associate with TEAD or other transcription factors to activate cell proliferation and inhibit apoptosis [61]. This signaling cascade is considered the core or downstream part of the Hippo pathway (Figure 1). Upstream from MST 1/2, the Hippo pathway does not have a single pathway; it is regulated by a large number of signals, such as cell polarity proteins, cell density, and mechanical forces [62]. In Drosophila, cells with apicobasal polarity have the protein complex located in their apical region, upstream of Warts (LAST in vertebrates), formed by kibra, Merlin (NF2 in vertebrates), and Expanded (Ex) [63]. Kibra and Merlin bind and phosphorylate Warts, preventing their ubiquitination and subsequent degradation resulting in the activation of these kinases leading to phosphorylation and inactivation of Yorkie (YAP and TAZ in vertebrates) [64]. This mechanism is Hippo-independent (MST 1/2 in vertebrates) and appears to be conserved in some cell types in vertebrates [65]. In invertebrates and vertebrates, MST 1/2 interacts with the STRIPAK complex containing the sarcolemmal membrane–associated protein (SLMAP) [66]. STRIPAK is a large protein complex formed by a catalytic subunit PP2A and the regulatory subunit Striatin (STRN) that recruits other proteins, including the SLMAP protein. STRIPAK complex interacts with MST 1/2, activating the Hippo pathway [66]. On the other hand, evidence shows that, in HEK293A cells, MST 1/2 is not essential for the activation of the pathway, but LATs 1/2 is. MST 1/2 deletion in 293T cells did not abolish YAP phosphorylation, whereas it is completely abolished in LATS 1/2 deleted cells. A screening to identify candidates that directly phosphorylate LATS 1/2 identified that MAP4K family kinases are responsible for phosphorylating LATS 1/2 acting in parallel with MST 1/2 [67].

Several reports, however, have provided substantial evidence indicating that cellular mechanotransduction is also a crucial factor in the regulation of YAP and TAZ. Importantly, and with few exceptions, this regulation was mostly described as capable of occurring independently of LATS 1/2 [21,68]. What is remarkable is that YAP/TAZ are capable of interpreting a wide range of mechanical signals and converting them into specific biological effects tailored to each cell type and mechanical stress. For example, in cells with low levels of mechanical signaling, such as rounded cells attached to a soft ECM (typically less than 1.5 kPa for epithelial cells) or cells attached to a small adhesive area (approximately 300 μm^2^), YAP/TAZ are primarily localized in the cytoplasm [21,68]. Conversely, in cells experiencing high levels of mechanical signaling, such as those cultured on rigid substrates (usually over 5–10 kPa) or subjected to increased cytoskeletal tension (cells spread over an area exceeding 3000 μm^2^), YAP/TAZ are predominantly found in the nucleus [21,68]. In addition, YAP/TAZ are subject to mechanical regulation through various forms of deformation, such as those originating from epithelial monolayers or the deformability of the ECM that supports cellular outgrowths [21,68,69]. In these contexts, YAP/TAZ not only senses cell and tissue mechanics but also play a crucial role in mediating the biological effects associated with mechanical signaling. This mechanotransduction process of YAP/TAZ has mostly been described as dependent on the actomyosin cytoskeleton, particularly on the specific organization of the F-actin cytoskeleton and on the contractile forces of non-muscle myosin II [21,70] (Figure 1). YAP/TAZ signaling can also be modulated by integrins, adherens junctions, other adhesion proteins, and GTPases, which connect the extracellular mechanical environment to the actomyosin cytoskeleton [71,72,73]. However, a crucial aspect that remains unsolved in the field is the connection between actomyosin architecture and the localization and activation of YAP/TAZ. Efforts in this direction have shown that a key step that allows YAP/TAZ to enter the nucleus is the opening of nuclear pores through nucleo-cytoskeletal coupling [74] (Figure 1). In addition, particularly regarding GTPases, Meng and colleagues [73] demonstrated that a Ras-related GTPase known as RAP2 serves as an intracellular signal transducer, transmitting signals of ECM rigidity to control mechanosensitive cellular activities through YAP/TAZ. Mechanistically, the authors showed that matrix stiffness exerts its influence through phospholipase Cγ1, affecting the levels of phosphatidylinositol 4,5-bisphosphate and phosphatidic acid, activating RAP2 via RAPGEF2 and RAPGEF6. When stiffness is low, active RAP2 binds to and stimulates MAP4K4, MAP4K6, MAP4K7, and ARHGAP29, consequently leading to the activation of LATS1/2 and inhibition of YAP/TAZ [73]. The signaling cascade described above integrates mechanical signals with LATS 1/2 and YAP/TAZ, differently from what has been widely described, although there is yet no established consensus in the literature on this subject (Figure 1).

The Hippo pathway is also regulated by cell density and cell-to-cell contact. When epithelial cells reach confluence in culture, the Hippo pathway is activated, and YAP leaves the nucleus and is located in the cytoplasm, suspending cell division. Unlike the control of the cytoskeletal stress and tension fiber-dependent pathway, the control of the Hippo pathway by cell density is carried out by the cascade involving activation of MST 1/2 and LAST 1/2 kinases [75].

The ability of YAP/TAZ to respond to these diverse mechanical inputs highlights their pivotal role as universal mechanotransducers and mechanoeffectors. Moreover, their involvement offers novel perspectives into the interpretation and comprehension of fundamental elements of tissue physiology and pathology at a molecular level. In this context, the identification of YAP and TAZ as mechanotransducers is finally uncovering the contribution of abnormal cell mechanics to the development of various diseases, such as cancer.

In this sense, there are currently few therapy strategies to directly and/or indirectly target the YAP/TAZ signaling (Figure 1). A detailed description of such strategies was elegantly reviewed in [76]. Verteporfin was the first compound discovered to inhibit YAP-TEAD binding and reduce organ overgrowth, although it is not an ideal clinical inhibitor due to its limited efficacy and unrelated effects. Other compounds that inhibit YAP-TEAD association, such as IAG933 by Novartis (NCT04857372), are currently in phase I clinical trial. Another way to inhibit the YAP/TAZ activity is by targeting YAP/TAZ expression directly by using antisense nucleotide methods. In this sense, ION537 by Ionis Pharmaceuticals (NCT04659096), currently in phase I clinical trial, has demonstrated great efficiency. Targeting TEAD directly to inhibit or disrupt YAP–TEAD complex is also under consideration. An example of such an alternative is VT3989, a compound developed by Vivace Therapeutics and also in phase I clinical trial (NC04665206) (Figure 1). Some drugs indirectly inhibit YAP/TAZ by targeting their upstream regulators, while others affect downstream molecules. For example, statins inhibit YAP nuclear translocation and enhance sensitivity to certain cancer drugs [77], while inhibitors of the BRD4 proteins are also under test, but the available data are insufficient to make conclusions for the moment [78]. Statins have also demonstrated an inhibitory effect on the growth of tumor xenografts, although their effectiveness appears to be more pronounced in vitro than in vivo. Some epidemiological studies have suggested that patients undergoing statin therapy have a reduced risk of developing cancer, although conflicting findings exist, highlighting the need for further investigations to evaluate the potential of these drugs in cancer treatment and prevention [77]. Targeting the regulation of YAP/TAZ by the cytoskeleton holds promise as a potential approach for developing anti-cancer therapies. Studies have indicated that the mechanical activation of YAP/TAZ in cancer cells plays a role in the development of chemoresistance [79]. However, the toxicity associated with anti-cytoskeletal treatments limits current therapeutic options (Figure 1). Thus, continuous efforts are still needed to develop other more efficient inhibitors of the YAP/TAZ/TEAD complexes, although promising ones are already under evaluation.

## 4. Hippo Signaling in GBM

Due to its role in controlling the balance between cell proliferation, apoptosis, resistance to chemotherapy, and metastasis [80,81], the Hippo pathway has been extensively studied over the years in various types of tumors. In the developing brain, the Hippo pathway effector, YAP1, is expressed in regions known as neural stem cell niches, the subventricular zone, and the external granular cell layer of the cerebellum [82]. In neural precursors of the granular layer of the cerebellum (Cerebellar granule neural precursors), the Sonic Hedgehog (Shh) pathway induces the nuclear localization of YAP that stimulates the proliferation of these cells [83]. Histological staining of samples from several types of astrocytomas demonstrated that YAP nuclear staining is common in almost all, including GBM, but uncommon in pilocytic astrocytoma [82]. TAZ is also overexpressed in GBM and stimulates cell proliferation and migration, and decreases apoptosis [84]. Xu and colleagues demonstrated that CD44 depletion in glioblastoma cells is responsible for activating the Hippo pathway, inhibiting tumor growth in vivo and in vitro, as well as sensitizing glioblastoma to temozolomide treatment [85]. CD44 binds to Merlin/NF2, and this interaction inhibits the binding of CD44 to HA. Merlin is downregulated in GBM, favoring CD44 binding with HA. Merlin/NF2 overexpression induces the activation of MST 1/2 and LATS 1/2, activating the Hippo pathway and inhibiting intracranial GBM growth [86].

The Hippo pathway in GBM also does not seem to follow a linear path, suggesting a large number of crosstalks with other signaling pathways. MST 1/2 depletion in GBM does not affect YAP activation but rather inhibits AKT and, consequently, mTOR [87]. The Hippo pathway is also involved in GBM response to radiotherapy. After treatment with ionizing radiation, the first response of GBM cells is the arrest of proliferation [88]. While the first response to ionizing radiation treatment involves the modulation of genes involved in apoptosis, cell cycle, and DNA repair, the long-term response involves genes that lead to cell senescence. One of the genes whose expression is inhibited by ionizing radiation is TAZ; however, this inhibition does not occur by the activation of LATS 1/2, but by inhibition of the WNT pathway. GBM cells treated with ionizing radiation have the WNT pathway inhibited and, therefore, the β-catenin destruction complex marks it for proteasome degradation. This same complex is responsible for the decrease in TAZ levels as a long-term effect of treatment [89]. Minata and colleagues demonstrated, however, that there is a difference between GBM stem cells located at the edge compared to those located in the center of the tumor. Cells of the invading edge preferentially express CD133, and when these cells are irradiated, they begin to express CD109, a marker preferentially expressed in the center of the tumor and leading to increased expression of YAP/TAZ target genes [90].

GBM has been categorized into four distinct molecular subgroups: proneural (PN), neural (NL), classical (CL), and mesenchymal (MES) [91]. Each subgroup is characterized by specific molecular alterations, response to therapy, and prognosis, making this classification crucial for guiding targeted treatment strategies. Among these subtypes, the MES subgroup is particularly aggressive and associated with a significantly worse prognosis compared to PN. Bhat and colleagues [92] provided compelling evidence demonstrating the role of TAZ in driving the MES phenotype observed in GBMs. These authors demonstrated that TAZ undergoes epigenetic silencing in PN GBMs, as compared to MES subtypes, and by manipulating TAZ expression in glioma stem cells and murine neural stem cells, they observed significant alterations in the expression of MES genes [92]. The findings implicate TAZ as a critical mediator in facilitating the transition toward the mesenchymal state in GBM.

**Figure 2:**
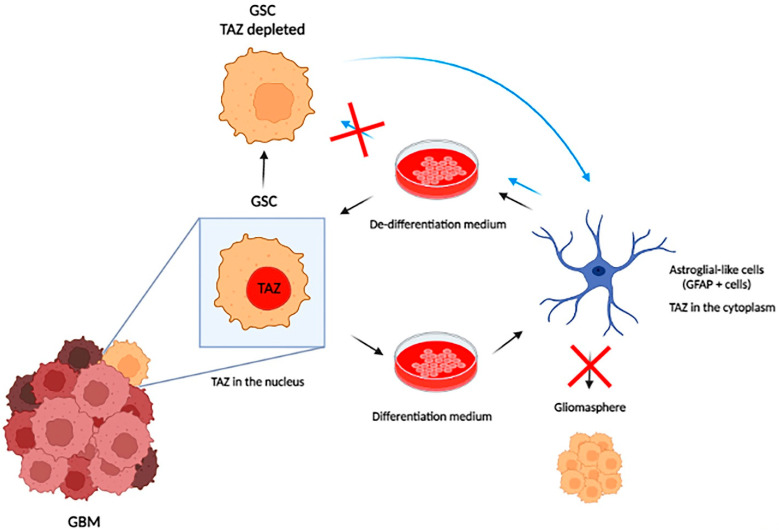
YAP/TAZ are key elements for maintaining the stem cell initiator state within GBM. GBM stem cells have an intrinsic plasticity that allows them to differentiate into astroglial cells or to express stem markers depending on the culture medium used (differentiation medium or de-differentiation medium). YAP/TAZ inhibition maintains glioblastoma cells in a differentiated state without returning to the stem state, even when placed in a de-differentiation medium. These differentiated cells are not capable of forming gliomaspheres and are less aggressive, preventing tumor progression.

YAP and TAZ are also required for the transformation of astrocytes (Figure 2). Mouse newborn astroglial cells were transfected with different oncogenes, and all induced activation of the YAP/TAZ reporter plasmid (8xGTIIC-RFP-DD). These same oncogenes are not able to induce the formation of oncospheres in astroglial cells depleted of YAP and TAZ [93]. These oncospheres were able to form a tumor mass with similar glioblastoma characteristics when implanted in a mouse brain with strong activation of TAZ in perinecrotic areas, as previously described [93]. Moreover, HER2 overexpression in astroglial cells induces the downregulation of astrocyte markers and upregulation of neural stem cell markers [93] in a YAP/TAZ-dependent manner. Taking advantage of a plastic characteristic of GBM cells, Castellan and coworkers tested the ability of Hu Tu 10 and Hu Tu 13 cell lines (primary GBM cell lines) to differentiate into an astrocytic phenotype by BMP2 induction or return to an undifferentiated state by placing cells in de-differentiation serum-free medium. Under these conditions, YAP/TAZ depletion prevented de-differentiation, and cells persisted with astroglial-like phenotypes even in the de-differentiation medium [93]. Taken together, these results show that YAP/TAZ are key factors in initiating the transformation of astroglial cells, and their inhibition can prevent one of the main obstacles to the treatment of glioblastoma, which is the presence of cells with stem phenotypes that are resistant to treatment (Figure 2).

## 5. Conclusions and Perspectives

Altogether, several studies have demonstrated that YAP and TAZ are activated in GBM and promote tumor growth and invasion. Thus, targeting YAP/TAZ signaling emerges as a promising strategy for GBM therapy. Several small molecules and peptides have been developed that inhibit YAP and TAZ activity [76,94] and could be used in combination with other therapies, such as radiation or chemotherapy, to treat GBM. Further research is needed to fully understand the mechanisms of YAP and TAZ regulation in GBM and to develop more effective YAP/TAZ direct or indirect inhibitors.

Another possible therapeutic approach is to manipulate the mechanical properties of the microenvironment to create anti-GBM conditions [95,96,97]. This approach may allow for the development of “smart” therapies that can locally or globally change mechanical properties to target the tumor [95,96,97]. Bioengineering strategies have been used in many studies to create a microenvironment-mimetic substrate to alter physical forces felt by cells, resulting in the elimination of malignant properties. In the future, this concept could be applied as a treatment by implanting engineered materials into tumor resection cavities after surgery, which respond to chemical and physical stimuli from surrounding brain tissue [98,99,100]. However, it requires further study and is yet to be developed for clinical application in GBM. Additionally, a more robust understanding of tissue mechanics together with other mechanosignaling pathways, such as those from Piezo1 ion channels and/or membrane tension, can also help pave the way for designing novel therapies that can mitigate biophysical barriers to other treatments, such as drug delivery or immunotherapy-based approaches [101,102,103]. Exploring the mechanical characteristics of the GBM microenvironment is highly promising. As we gain more insights into the mechanobiological aspects of the microenvironment, we can better comprehend the disease progression, which can lead to innovative therapeutic and diagnostic opportunities, as well as better preclinical modeling to come close to a cure for this very aggressive tumor.

## Data Availability

Data sharing is not applicable to this article.
